# West Nile virus encephalomyelitis in an immunocompromised patient

**DOI:** 10.1016/j.radcr.2023.09.050

**Published:** 2023-10-13

**Authors:** Abdulmunaim M. Eid, Mohammad Obadah Nakawah

**Affiliations:** a Stanley H. Appel Department of Neurology, Houston Methodist Neurological Institute, 6560 Fannin St. Scurlock Tower, Suite 750, Houston, TX 77030, USA

**Keywords:** Acute leukoencephalopathy, Encephalomyelitis, Neuroimaging, West Nile virus

## Abstract

We report a case of a 50-year-old immunocompromised woman who presented with acute encephalopathy and gait ataxia due to severe West Nile virus encephalomyelitis. The patient remarkably improved following early and sustained aggressive immunomodulation. Diagnosing West Nile neuroinvasive disease could be challenging without biochemical evidence of West Nile virus nucleic acid in the cerebrospinal fluid, a specific but not sensitive test. Although the neuroimaging findings in our patient could be considered “typical” for West Nile neuroinvasive disease, there is an overlap with the imaging abnormalities seen in other neuroinflammatory disorders presenting with acute leukoencephalopathy. Hence, we review West Nile neuroinvasive disease imaging characteristics and the differential diagnosis of acute leukoencephalopathy.

## Introduction

Magnetic resonance imaging (MRI) in West Nile neuroinvasive disease (WNND) classically shows bilateral lesions in parasagittal structures, particularly the brainstem, and thalamus, but also the basal ganglia, cerebellum, hemispheric white matter, and temporal lobe [1]. These lesions are typically hyperintense on T2-weighted images (T2WI) and hypointense on T1-weighted images (T1WI), with variable degrees of free or restricted diffusion. This MRI signature, however, is not specific to WNND but rather is shared among many inflammatory, vascular, toxic, and metabolic neurological disorders that present with acute leukoencephalopathy.

## Case description

**Fig. 1:**
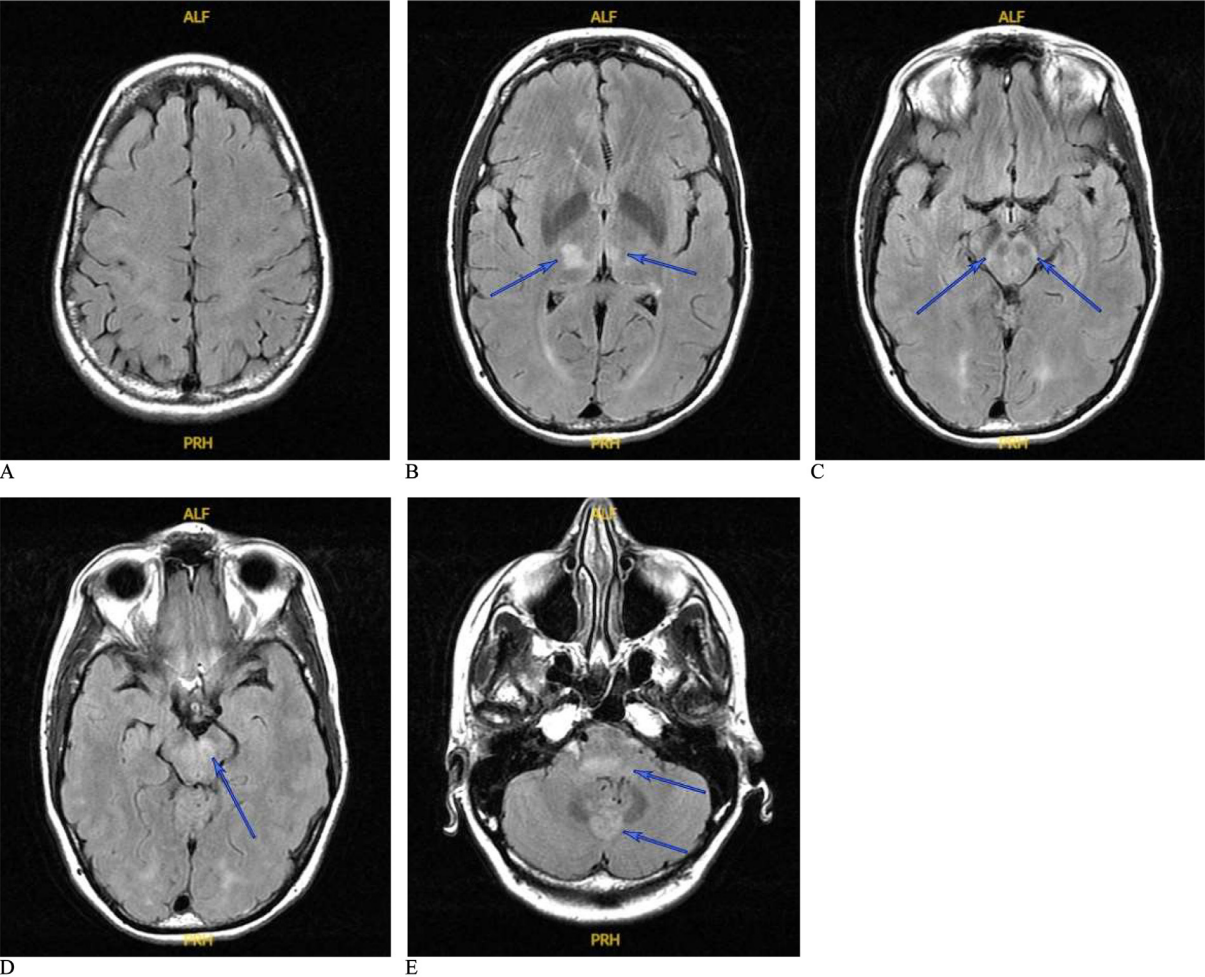
(A) Brain MRI on post-admission day 3. Multiple axial slices of T2-FLAIR (right) show hyperintense lesions in the bilateral posterior thalami (right > left; B), the midbrain, particularly abutting the red nuclei (C, D), the posterior pontomedullary region and cerebellum (E). There is no mass effect or atrophy out of proportion to expected volume changes at this age. No diffusion restriction was evident (not shown), and no Gd study was performed.

**Fig. 2:**
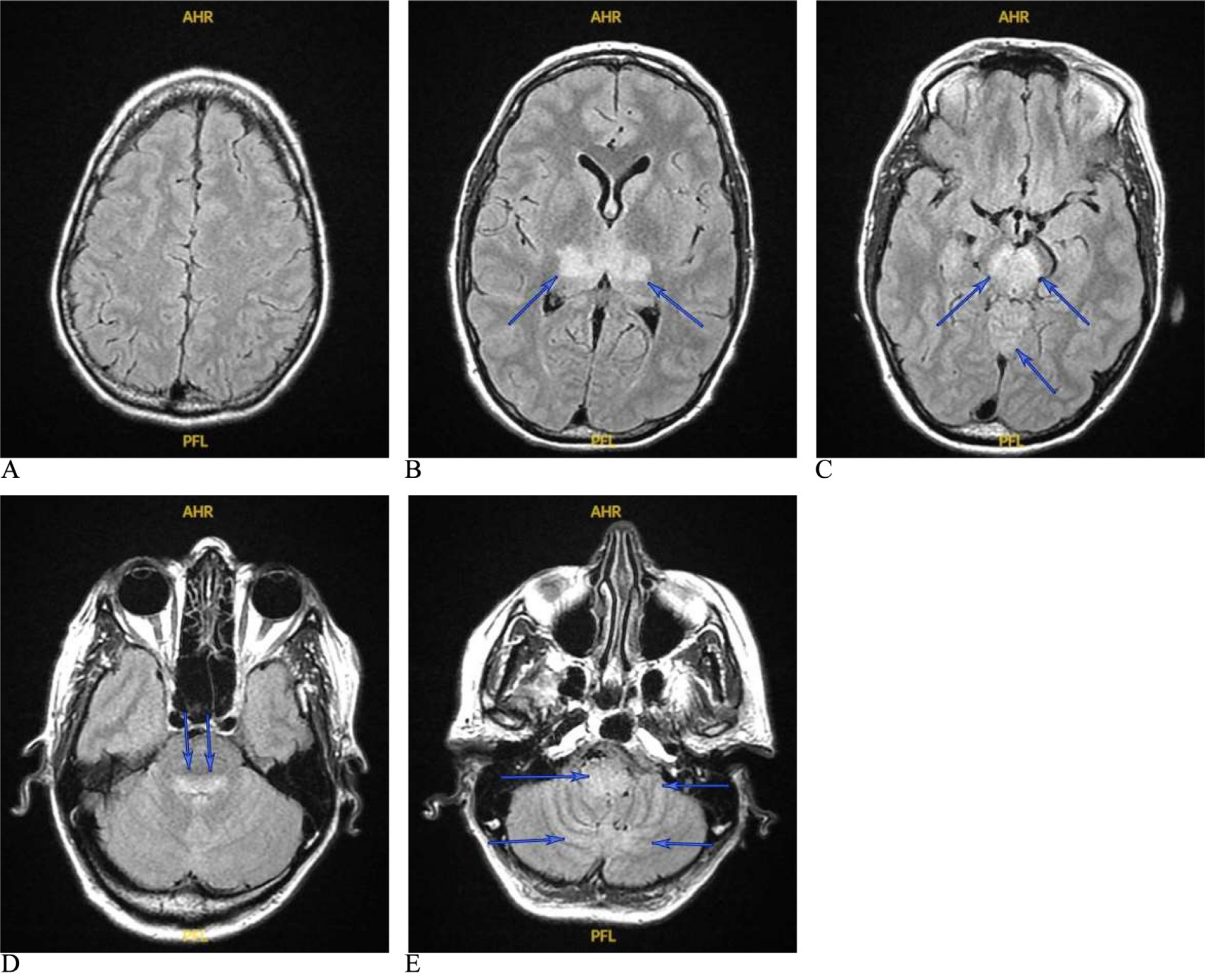
(A) Brain MRI on post-admission day 4. Multiple axial T2-FLAIR slices show interval progression of the hyperintense lesions in the bilateral posterior thalami (B), the midbrain (now involving a larger area; C), the posterior pons (D), and the posterior medulla and cerebellum (E). Corresponding areas of hypointense signals are noted on T1WI (not shown). There was no evidence of diffusion restriction (not shown), and no Gd study was performed.

**Fig. 3:**
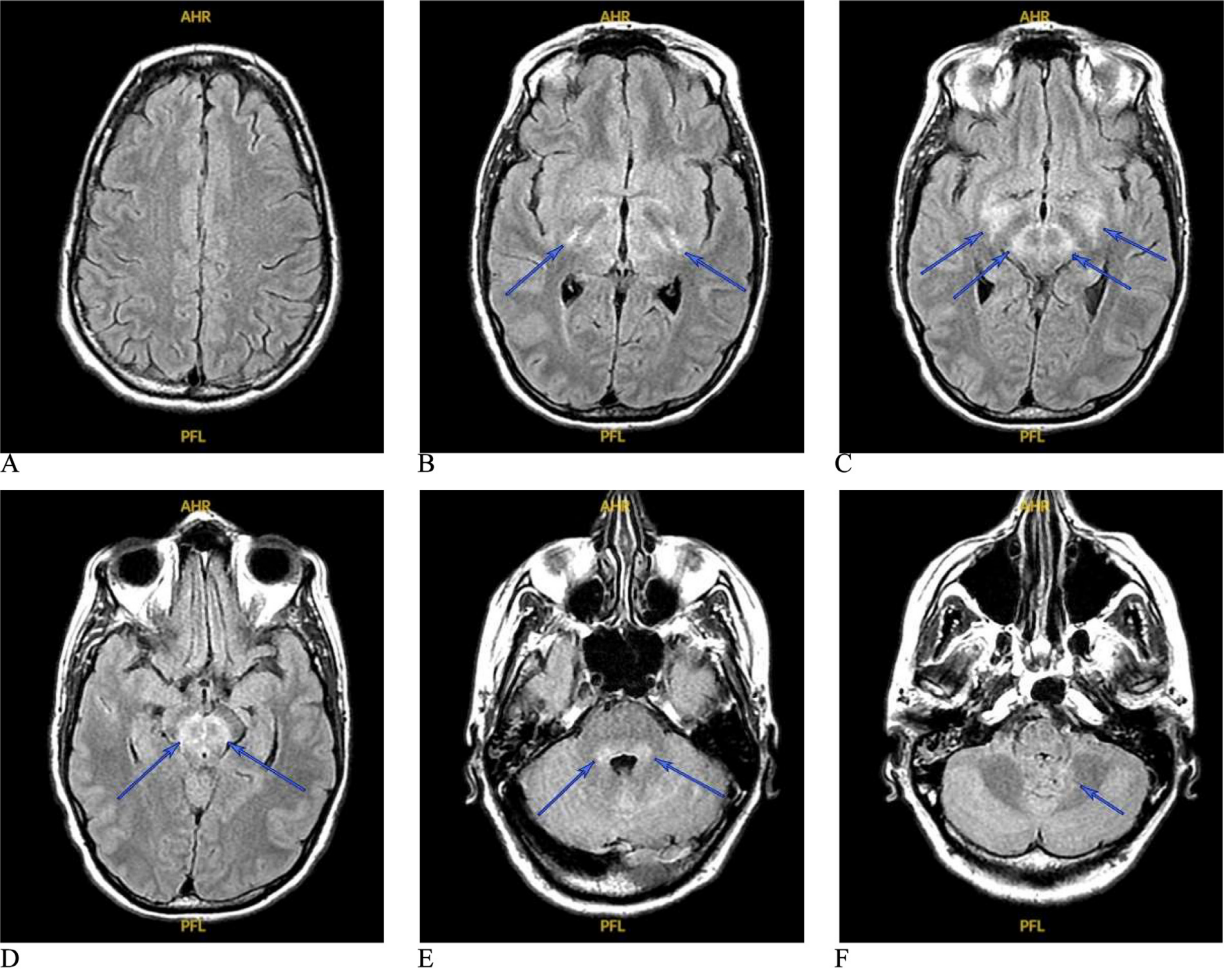
(A) Brain MRI on post-admission day 14. Multiple axial slices of T2-FLAIR show modest improvement in the signal changes in the posterior thalamus and the brainstem on post-admission day 14 despite new mild involvement of the posterior basal ganglia (B).

**Fig. 4:**
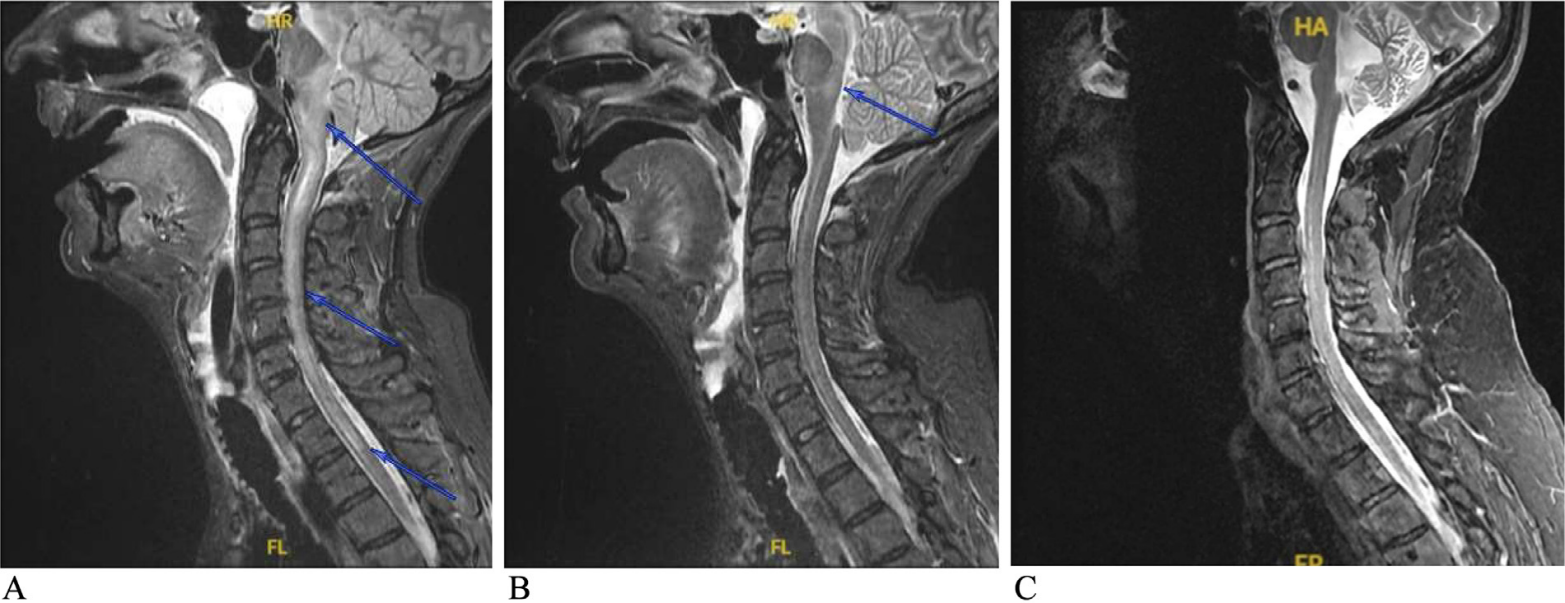
Cervical spine MRIs on post-admission day 3 (A), post-admission day 14 (B), and 4.5 years later (C). Sagittal STIR images are shown. There is a longitudinally extensive spinal cord STIR hyperintense lesion spanning C2-C8 segments and extending rostrally to the brainstem (A) that greatly improved on the subsequent scan (B). Sagittal STIR 4.5 years post-admission reveals marked cerebellar vermis atrophy without signal abnormalities possibly secondary to degeneration of the tracts connecting cerebellum to affected brainstem and spinal cord regions (diaschisis).

A 50-year-old right-handed Caucasian woman with a remote history of kidney transplantation due to IgA nephropathy, on tacrolimus and mycophenolate for several years, presented to our hospital with 7 days of fever, myalgia, rash, vomiting, diarrhea, intermittent headache, neck stiffness, mild confusion, gait instability, and hands tremor. The initial examination revealed low-grade fever, maculopapular rash on trunk and limbs, and resting and kinetic hands tremor consistent with Holmes tremor. Brain MRI without gadolinium (Gd), obtained on day 3 after admission, showed abnormal T2 fluid-attenuated inversion recovery (FLAIR) hyperintensities in the thalami and brainstem (Fig. 1); contrast agents were avoided due to a history of anaphylactic reaction to Gd-based contrast media. Cerebrospinal fluid (CSF) analysis revealed lymphocytic pleocytosis and a positive qualitative polymerase chain reaction (PCR) for WNV, while WNV IgM and IgG levels were normal in both CSF and serum. A diagnosis of acute WNV encephalitis was made. On day 4, the patient developed worsening confusion, gait ataxia, and Holmes tremor, as well as dysarthria, bilateral down-beating nystagmus, and truncal titubation. A repeat brain MRI showed interval extensive progression of the T2-FLAIR hyperintensities throughout the thalami, cerebral peduncles, dorsal pons, and dorsal medulla (Fig. 2). Cervical and thoracic spine MRI showed extensive STIR hyperintensities throughout the cervical and upper thoracic cord (Fig. 4). The patient developed acute respiratory failure and seizure-like activity, warranting endotracheal intubation for ventilatory support. Continuous electroencephalogram (EEG) monitoring revealed no epileptiform activity. The doses of tacrolimus and mycophenolate were decreased, and she was placed on intravenous immunoglobulin (IVIG) 0.4 g/kg/day (25 g daily) × 5 days, followed by methylprednisolone 1000 mg IV daily × 5 days. After a few days, the patient slowly became more responsive and was able to follow very simple verbal commands. A repeat brain and cervical spine MRI were obtained on day 14 after admission, showing interval improvement of the T2-FLAIR hyperintensities, especially in the lower brainstem and the cervical spine (Figs. 3 and 4B). CSF studies 30 days after admission revealed more prominent lymphocytic pleocytosis, which was believed to represent immune reconstitution. Although CSF qualitative WNV PCR was negative this time, WNV IgM and IgG antibodies in the CSF were elevated. Due to persistent neurological dysfunction, she received another course of IVIG and high-dose IV methylprednisolone, followed by a slow oral prednisone taper (80 mg daily decreased by 10 mg weekly). Amantadine 100 mg twice daily was used for the symptomatic control of Holmes tremor and truncal titubation, while levetiracetam 1000 mg twice daily was used for seizure prevention. The patient was subsequently transferred to acute inpatient rehabilitation on day 45, followed by outpatient physical therapy for several months.

Five months after the initial presentation, the patient's cognitive function returned to baseline. However, she had hypophonia, vertical nystagmus, diffuse muscle weakness with mild hypertonia in all limbs, cervical dystonia, head tremor, and resting and kinetic intention hands tremors. She was able to stand up with assistance but mostly used a wheelchair. Brain and cervical spine MRI then showed near-complete resolution of the T2-FLAIR signal abnormalities (not shown). CSF studies also showed marked improvement in lymphocytic pleocytosis. Monthly IV methylprednisolone 1000 mg for 6 months was initiated to address the potential persistent low-grade CNS inflammation. Twelve months following her initial presentation, the patient's truncal titubation, head tremor, and nystagmus have all resolved. CSF analysis at that time showed resolution of the lymphocytic pleocytosis. Amantadine and levetiracetam were discontinued gradually. Her motor function improved significantly over the following years except for moderate gait ataxia and action hands tremor which could be attributed to the development of cerebellar atrophy (Fig. 4C).

## Discussion

WNV is one of the most common arboviruses in the world but had not been detected in the western hemisphere until a cluster of WNV meningoencephalitis emerged in New York City in 1999 [1], [2]. Since that time, there have been more than 56,000 cases of WNV infections reported to the US Centers for Disease Control and Prevention (CDC); approximately 50% of cases were neuroinvasive infections [3], [4]. The mortality rate is 5%, but nearly all deaths (95%) accompany the neuroinvasive form [3], [4]. Risk factors include advanced age, diabetes, alcohol abuse, and immunosuppression [5]. The clinical presentation of our patient reflects the typical description of WNND in the medical literature, including fever, myalgia, gastrointestinal disturbances, maculopapular rash, confusion (meningoencephalitis), and acute flaccid paralysis (poliomyelitis-like illness) [6], [7]. The patient also had Holmes tremor, which emerges from dysfunction of the dentato-rubro-thalamic circuitry and the nigrostriatal tracts [2], [8], [9]. The patient's limb and gait ataxia, rigidity, head tremor, and titubation suggest involvement of structures contributing to these circuits as well. Brain MRI showed clear involvement of the posterior thalamus and most of the midbrain, especially the substantia nigra and red nucleus (most conspicuous in Fig. 2). Tremors, parkinsonian signs (rigidity, bradykinesia, postural instability) and cerebellar signs (dysmetria, cerebellar nystagmus, gait ataxia) are reported in WNND in up to two-thirds of cases [8], [10], [11]; all of which were present in our patient. WNND can be associated with weakness secondary to myelitis, poliomyelitis-like disease (ie, anterior horn cells and/or anterior root involvement), or Guillain-Barre syndrome [12]. The extensive initial involvement of the spinal cord seen on MRI suggests severe myelitis (Fig. 4A).

CSF analysis in WNND shows pleocytosis (with either lymphocytic or polymorphonuclear predominance) in ≥96% of cases [13]. Our patient had lymphocytic pleocytosis since early in the disease course. In our patient, CSF cell count significantly increased from 31 to 272 WBCs in 4 weeks despite improvement in the patient's clinical condition. This is atypical as 94% of patients show decreased CSF cell count between the first and the second CSF examinations (median time between the 2 examinations = 4 days, range = 1-18 days), while the minority of patients show stable or slightly increased cell count [13]. We attribute this atypical CSF finding in our case to immune reconstitution syndrome since the doses of long-term immunosuppressants were decreased midway between the 2 lumbar punctures to allow for improvement in the patient's immune response. Although WNV PCR testing in the CSF is relatively insensitive (57%-70%), it is 100% specific for the diagnosis [14], which facilitated an early confirmed diagnosis in our patient. The detection of antibodies in the CSF can facilitate the diagnosis when PCR is negative, but these assays could detect heterologous cross-reacting antibodies from other viruses and could be falsely positive in rheumatologic diseases [15]. Furthermore, the presence of IgM antibodies, which indicates an acute or “recent” infection, could be delayed (such as in our patient's case when they were only detected on the second CSF examination) and could persist for more than a year rendering it less useful for diagnostic and monitoring purposes [16], [17]. This makes neuroimaging diagnostically useful, particularly when CSF PCR is negative or not available, as we will discuss.

MRI studies show abnormalities in 20%-70% of WNND cases [1], [7], [8], [18], [19], [20]. The variation could be due to the type of sequences used and the timing of acquiring the scans. The most common is bilateral T2-FLAIR hyperintense lesions in parasagittal structures, particularly the brainstem (38.9%) and thalamus (33.3%), with less prevalence in the basal ganglia (5.6%), cerebellum (5.6%), hemispheric white matter (5.6%), and temporal lobe (5.6%) [1]. As seen in Figs. 1-3, our patient had bilateral T2-FLAIR hyperintense lesions in the thalami, midbrain, pons, and medulla. There was a faint hyperintense signal in the peri-rolandic white matter bilaterally in the first scan (Fig. 1), but this disappeared in subsequent scans (Figs. 2-4), unlike the other lesions, which worsened, then subsequently improved. Diffusion restriction, a finding absent in our patient (imaging not shown), could be seen in WNND lesions in up to 50% of cases [7], [20]. The lesions are typically isointense on T1WI [1], [19], [21], but we noticed a hyperintense signal of the cerebral peduncles and potentially the basis pontis that could be true hyperintensity of these structures or relative hypointensity of the adjacent substantia nigra (the former possibility is more likely). These parenchymal lesions do not typically enhance with Gd [1,^19^,21]. Most patients with WNND have normal spine MRI scans but abnormalities involving the roots, cauda equina, conus, or the anterior cord are reported [1], [8], [19], [20], [21], [22]. Systemic studies are absent, but the presence of cord signal changes seems the least frequent, even in patients with acute flaccid paralysis suspicious for myelitis [22]. Our patient had an extensive longitudinal hyperintense anterior cord lesion spanning C1 to T2, most conspicuous on sagittal short tau inversion recovery (STIR) (Fig. 4).

The lesions observed on MRI in our case primarily involved white matter structures (eg, cerebral peduncles, dorsal pons, and dorsal medulla), but also, to an extent, deep gray matter structures, particularly the thalamus. This pattern can be seen acutely in multiple neurologic disorders and could be generally referred to as “acute leukoencephalopathy.” Acute leukoencephalopathies are associated with often symmetric white matter lesions that can be either transient (such as in our case) or progressive [23], but gray matter structures are often affected to a much lesser degree. In the remainder of this section, we will discuss relatively common disorders presenting with acute leukoencephalopathy from a neuroimaging perspective.

### 1) Inflammatory and demyelinating disorders

*Acute disseminated encephalomyelitis (ADEM)* is a severe immune-mediated inflammatory disorder of the CNS that predominantly affects the white matter of the brain and spinal cord. ADEM MRI lesions are often large, patchy, and poorly marginated in morphology. They typically asymmetrically involve the subcortical white matter and cortical gray-white junction, brainstem, cerebellum, and spinal cord [24]. In addition, thalamic and basal ganglia involvement is often seen [24]. Gd enhancement occurs in 14%-30% of cases [25]. Although ADEM is usually a monophasic disease, new lesions may be seen on follow-up MRI within 3 months after the onset of symptoms. When the spinal cord is involved, the thoracic region is the typical location [24]. The use of MRI to demonstrate lesions dissemination in both time and space, as emphasized by the revised McDonald criteria [26] increases the sensitivity of *multiple sclerosis (MS)* diagnosis but does not necessarily increase its specificity. Typical MS lesions are hyperintense on T2WI, while active lesions usually enhance with Gd and/or have restricted diffusion. The perivenular pattern of lymphocytic infiltration gives rise to the characteristic distribution of lesions T2-FLAIR sequences that are radially oriented to the lateral ventricles. *Neuromyelitis optica spectrum disorder (NMOSD)* is a spectrum of autoimmune disorders that classically affect the optic nerve and spinal cord. However, this classic view has long been abandoned since several studies showed that 50%-85% of patients have brain lesions as well [27]. Areas with high aquaporin 4 (AQP4) expression are affected most; these are adjacent to the ventricular system at any level, such as the corpus callosum, hypothalamus, peri-ependymal areas surrounding the third and lateral ventricles, cerebral aqueduct, and dorsal brainstem adjacent to the fourth ventricle [27]. Antibodies against myelin oligodendrocyte glycoprotein (MOG-IgG) could cause optic neuritis, myelitis, and brainstem encephalitis, as well as ADEM-like presentations [28], [29], known as *MOG antibody disease (MOGAD)*. Longitudinally extensive lesions of the spinal cord (3 or more contiguous segments) and longitudinally extensive involvement of the optic nerve (often bilateral) are quite distinctive for both NMOSD and MOGAD. Diencephalic and middle cerebellar peduncle lesions could be seen in either. Typical acute lesions have a high signal on T2WI and enhance with Gd. Bilateral perineural optic sheath enhancement and cortical leptomeningeal enhancement are seen in MOGAD [30].

### 2) Toxic-metabolic and hypoxic encephalopathies

*Several toxic-metabolic encephalopathies* reflect metabolic alterations secondary to cardiac, pulmonary, or hepatic dysfunction; in such instances, the imaging pattern is similar to that of *hypoxic-ischemic encephalopathies*, that is, there is a predilection to the gray matter, particularly in the depth of the cortical sulci, globus pallidus, and the superior portion of the cerebellar cortex [23]. This pattern that follows the gray matter is unique and, paired with the acute presentation, could be enough for a broad diagnosis. Specific examples include carbon monoxide poisoning and cyanide poisoning. Interestingly, a delayed leukoencephalopathy may rarely follow hypoxic-ischemic events, for example, from carbon monoxide poisoning [31]. In these cases, the initial event is clinically mild and brief with no imaging findings, but a few days or weeks later, there is clinical worsening and leukoencephalopathy on MRI [31]. The entity is called “delayed post-hypoxic leukoencephalopathy.” Other toxins characteristically damage the basal ganglia, for example, manganese toxicity (either from inhalation of manganese fumes or liver failure) which characteristically causes T1 hyperintensity in the globus pallidus, and, to some extent, putamen, thalamus, midbrain, and hippocampus [32]. The T1 hyperintensity in the globus pallidus could happen in liver failure (from any etiology), basal ganglia mineralization, or hyperalimentation, as a consequence of a prior hypoxic-ischemic event or from retained Gd contrast [23]. Another toxin is methanol which is metabolized into formate—a cytotoxic substance that interferes with intramitochondrial electron transport and particularly affects the putamen (causing necrosis and hemorrhage) and possibly the subcortical white matter [33], [34]. The imaging appearance is characteristic; bilateral mixed FLAIR signal in the putamen, T2* hypointensity reflective of hemorrhage, and a higher signal on T2-FLAIR and T1 with Gd of the periphery of the lesion giving the characteristic “lentiform fork sign” [33], [34]. Wernicke's encephalopathy leads to T2-FLAIR hyperintense lesions in the mammillary bodies and the gray matter surrounding the third ventricle, cerebral aqueduct, and fourth ventricle [35]. Gd enhancement is usually seen. In *osmotic demyelination syndrome*, rapid correction of severe hyponatremia or severe hyperosmolality causes demyelination that appears hypointense on T1WI and hyperintense on T2WI, often with diffusion restriction [36]. Severe lesions may enhance with Gd [36]. Although it most commonly affects the basis pontis, other sites such as the cerebellum, thalami, especially the lateral geniculate body, basal ganglia, external capsule, extreme capsule, hippocampi, and even the gray-white junction in the cerebral cortex are not infrequently affected [36]. *Cytotoxic lesions of the corpus callosum (CLOCCs)* is another entity that is characterized by acute transient leukoencephalopathy, classically seen in patients with active seizures or metabolic disturbances. However, there is a plethora of etiologies that could give rise to CLOCCs, including influenza viruses A and B, other infections, antiepileptic medications, high-altitude cerebral edema, hyperammonemia, hypernatremia, hypoglycemia, antidepressants, and chemotherapy. The corpus callosal (often splenial) lesions are hyperintense on T2-FLAIR, have homogenous diffusion restriction, and are typically reversible [37]. In high-altitude cerebral edema, diffusion restriction may be absent suggestive of vasogenic rather than cytotoxic edema [37]. Despite the name, some patients may have other white matter involvement outside of the corpus callosum. The lesions do not enhance [37]. With metronidazole encephalopathy, the splenial lesion could be accompanied by periaqueductal gray and dentate nucleus involvement [38].

### 3) Vascular disorders

*Posterior reversible encephalopathy syndrome (PRES)* is typically considered a form of acute leukoencephalopathy despite the involvement of gray matter in a sizeable portion of the cases (40% in one series) [39]. However, the white matter changes tend to be the most conspicuous. The syndrome is almost always accompanied by T2-FLAIR hyperintense lesions in the occipital and parietal lobes, but also frequently in the frontal and temporal lobes, basal ganglia, thalami, brainstem, and cerebellum [40]. Diffusion restriction and hemorrhage rarely occur [41], [42]. *Reversible cerebral vasoconstriction syndrome (RCVS)* can cause white matter infarcts, typically in a water-shed distribution [43]. *Multifocal cerebral arteriopathies* such as primary angiitis of the CNS (PACNS), varicella-zoster vasculitis, and human immunodeficiency virus (HIV) vasculitis could cause acute white matter infarctions that need to be differentiated from other causes of acute leukoencephalopathy. These infarctions span multiple vascular territories and would be less symmetric than other causes of acute leukoencephalopathy [44]. *Neuro-Behcet's disease* is a rare idiopathic vasculitis with predilection for the venular system. It can present with subacute meningoencephalitis causing lesions similar to WNND, including T2-hyperintense lesions in the periventricular white matter, basal ganglia, thalamus, brainstem, and cerebellum [45]. Venous infarctions, hemorrhage, and focal enhancement in the posterior globes suggesting uveitis could be differentiating imaging findings [45].

## Conclusion

This case highlights the broad differential diagnosis of acute leukoencephalopathy and the usefulness of neuroimaging in approaching this diagnostic dilemma if the definitive WNV PCR in the CSF is negative or unavailable. Furthermore, we demonstrated a clear association between the patient's clinical symptoms and the MRI lesions load and locations.

## Patient consent

Written informed consent for publication of the case was obtained from the patient.
